# Effects of Spike Anticipation on the Spiking Dynamics of Neural Networks

**DOI:** 10.3389/fncom.2015.00144

**Published:** 2015-11-30

**Authors:** Daniel de Santos-Sierra, Abel Sanchez-Jimenez, Mariano A. Garcia-Vellisca, Adrian Navas, Jose A. Villacorta-Atienza

**Affiliations:** ^1^Group of Biometrics, Biosignals and Security, Research Centre for Smart Buildings and Energy Efficiency (CeDInt), Technical University of MadridMadrid, Spain; ^2^Laboratory of Computational System Biology, Center for Biomedical Technology, Technical University of MadridMadrid, Spain; ^3^Department of Applied Mathematics (Biomathematics), School of Biological Sciences, Universidad Complutense de MadridMadrid, Spain; ^4^Department of Applied Mathematics, School of Mathematics, Universidad Complutense de MadridMadrid, Spain

**Keywords:** spike anticipation, information processing, neural networks, synchronization, chaotic dynamical systems

## Abstract

Synchronization is one of the central phenomena involved in information processing in living systems. It is known that the nervous system requires the coordinated activity of both local and distant neural populations. Such an interplay allows to merge different information modalities in a whole processing supporting high-level mental skills as understanding, memory, abstraction, etc. Though, the biological processes underlying synchronization in the brain are not fully understood there have been reported a variety of mechanisms supporting different types of synchronization both at theoretical and experimental level. One of the more intriguing of these phenomena is the anticipating synchronization, which has been recently reported in a pair of unidirectionally coupled artificial neurons under simple conditions (Pyragiene and Pyragas, 2013), where the slave neuron is able to anticipate in time the behavior of the master one. In this paper, we explore the effect of spike anticipation over the information processing performed by a neural network at functional and structural level. We show that the introduction of intermediary neurons in the network enhances spike anticipation and analyse how these variations in spike anticipation can significantly change the firing regime of the neural network according to its functional and structural properties. In addition we show that the interspike interval (ISI), one of the main features of the neural response associated with the information coding, can be closely related to spike anticipation by each spike, and how synaptic plasticity can be modulated through that relationship. This study has been performed through numerical simulation of a coupled system of Hindmarsh–Rose neurons.

## 1. Introduction

The nervous system in insects, animals, and humans has evolved to allow an accurate and versatile information processing adapted to their particular environments. However, despite the diversity of the cognitive abilities of living beings, the most evolved species share common neural mechanisms, both at neural and network scales, supporting information processing and coding (Kandel et al., 2000).

The central paradigm of neural information coding at cellular scale is the timing between consecutive action potentials or spikes (Koch, 1999). This interspike interval or ISI is the key to characterize the diverse activity regimes in real neurons and so the variety of information processing in the nervous system (Izhikevich, 2007), although subthreshold oscillations have also been proposed as a mechanism for coding neural information (Hänggi, 2002; Villacorta-Atienza and Panetsos, 2005).

These cellular mechanisms support information processing at network scale, where different functional processes appear to take advantage of the diversity and complexity of connective structures, transmission phenomena (as excitation–inhibition interplay or delay), etc. Among these processes synchronization has raised as one of the central brain mechanisms involved in high cognitive abilities (Varela et al., 2001). From a conceptual point of view synchronization is the coordinated behavior of several coupled dynamical systems, but this definition is specially natural in the field of neuroscience since it is known that the nervous system requires the coordinated activity of both local and distant neural populations. Many different types of synchronization have been described, both in deterministic and chaotic systems (Pikovsky et al., 2003). The most interesting and biologically-motivated classes of synchronization concern diverse features of chaotic dynamics, as complete synchronization (Pecora and Carroll, 1990), generalized synchronization (where dynamical features of the system are not equal but related by a functional dependence over time; Rulkov et al., 1995), phase synchronization (Rosemblum et al., 1996), and anticipating synchronization (Voss, 2000). In this last type of synchronization a driven element in a unidirectionally coupled chaotic system can synchronize its behavior in advance to the activity of the driving element.

Recently a new anticipating synchronization has been reported in unidirectionally coupled pair of oscillators (Pyragiene and Pyragas, 2013). In this type of anticipation the response of the slave (driven oscillator) precedes in time the behavior of the master (driving oscillator); for instance in the case of coupled neurons, the action potential, or *spike*, elicited by the slave neuron will occur before the appearance of the corresponding spike coming from the master neuron. The main finding of this reporting is that this anticipation appears under a single requirement: the mean frequency of the uncoupled slave must be greater than the mean frequency of the master. This finding improves previous results, where anticipating synchronization were reported theoretical and experimentally in multiple models and experimental setups but with important restrictions, as the necessity of memory elements in the master element or time-delay self-feedbacks in the slave oscillator (see Pyragiene and Pyragas, 2013 and references therein).

In this paper, we explore the anticipating synchronization introduced by Pyragiene and Pyragas, in the context of the spiking dynamics of neural networks and its plasticity, as a main functional mechanism of the network. We consider two complementary approaches, developed by means of numerical simulations. On the one hand, we study the impact of spike anticipation over the activity of a neural network exhibiting common features of complex theoretical and biological neural networks as closed loops, relay neurons, excitatory and inhibitory coupling, and convergent and divergent information pathways. The neural network firing activity is a direct reflection of its information processing, both from the basic information theory (Shannon and Weaver, 1959) and the information processing in the nervous system, which ranges from the simplest coding of the stimulus intensity through the increase of the firing frequency in the peripheral sensory neurons (Kandel et al., 2000) to the complex dynamical and statistical relationships between firing activity of different neural populations in high-level brain areas (Koch, 1999; Benito et al., 2014). On the other hand, we study the effects of spike anticipation over synaptic plasticity, responsible for the reinforcement/debilitation of connections among neurons, a critical aspect of information processing in the nervous system.

The main objective of our work is to characterize qualitatively the effects of spike anticipation over a variety of main dynamical and functional aspects of the neural networks related with their information processing. We remark the simplicity of the conditions required to exhibit such effects and therefore, their potential significance as a novel factor to be considered together with other well-known processes critical in information processing, as input excitatory/inhibitory balance, time-delay, spiking regime of individual neurons, network topology, etc.

## 2. Materials and methods

Throughout this paper we will consider the Hindmarsh–Rose neuron (HR, Hindmarsh and Rose, 1984) as a versatile neuronal model capable of exhibiting numerous features and behaviors typical of real neurons (Izhikevich, 2004). The unidirectional coupling of HR neurons with the proper parameters ensures the appearance of spike anticipation (Pyragiene and Pyragas, 2013), so the backbone of this work will be a basic network composed of a *master* neuron coupled to a chain of *n* intermediary neurons, whose last neuron is coupled to the *slave* neuron. This network is mathematically described by the system:

(1) $$\begin{array}{l} C_{m}{\dot{x}}_{m}=y_{m}+x_{m}^{2}(b-ax_{m})-z_{m}+J_{0},\\ {\dot{y}}_{m}=c-dx_{m}^{2}-y_{m},\\ {\dot{z}}_{m}=r(s(x_{m}-x_{st})-z_{m}),\\ ...\\ C_{i}{\dot{x}}_{i}=y_{i}+x_{s}^{2}(b-ax_{i})-z_{i}+J_{0}+k_{i}(x_{i-1}-x_{i}),\\ {\dot{y}}_{i}=c-dx_{i}^{2}-y_{i},\\ {\dot{z}}_{i}=r(s(x_{i}-x_{st})-z_{i}).\\ ...\\ C_{s}{\dot{x}}_{s}=y_{s}+x_{s}^{2}(b-ax_{s})-z_{s}+J_{0}+k_{s}(x_{n}-x_{s}),\\ {\dot{y}}_{s}=c-dx_{s}^{2}-y_{s},\\ {\dot{z}}_{s}=r(s(x_{s}-x_{st})-z_{s}), \end{array}$$

where the subscripts *m* and *s* will denote the *master* and *slave* neurons and *i* = 1, …, *n* is the index for the *n* intermediary neurons (*i* = 0 denotes the master neuron). The parameters with no these subscripts take the values of: *a* = 1, *b* = 3, *c* = 1, *d* = 5, *s* = 4, *r* = 0.005, *x*_*st*_ = −1.6, and *J*_0_ = 3.25. The membrane capacitances *C*_*m*_, *C*_*i*_, and *C*_*s*_ determine the oscillatory behavior of the neurons by modulating their time scale, exhibiting silence, subthreshold oscillations or “spiking” activity (when action potentials appear).

The spike anticipation will be quantified by the difference between the time corresponding to a local maximum of a certain master spike and the time corresponding to the local maximum of the nearest slave spike. Thus, a positive spike anticipation will denote a slave spike occurring previously to the driving master spike. In order to exhibit synchronization by spike anticipation the master, intermediary, and slave neurons must fulfill that *C*_*m*_ > *C*_1_ > … > *C*_*i*_ > … > *C*_*n*_ > *C*_*s*_, so each driven neuron (in uncoupled conditions) will be slightly faster than its corresponding driving neuron. In this study, we fixed these parameters to *C_m_* = 1 and *C*_*s*_ = 0.7, with ${\left\{C_{i}\right\}}_{i=1}^{n}$ equispaced in the interval (0.7, 1). Finally, the parameters ${\left\{k\right\}}_{i=1}^{n}$ and *k*_*s*_, all equal to 1.7, determine the coupling strength between the HR neurons in the system.

The dynamics of the system described by Equation 1 was simulated in Matlab (Matlab 2012a 64-bits, The Mathworks, Inc.) by means of the standard solver implementing the adaptive explicit Runge–Kutta method of degrees (4, 5). The initial conditions were fixed to zero and the chaotic network dynamics was considered after a transient time *t*_*tr*_ = 300, taking a time discretization of 0.01 units of time and during a time interval of *T*, where usually *T* = 5 × 10^4^. The results were obtained with a precision given by tolerances in the adaptive algorithm of 10^−10^ but they are robust against higher tolerances up to 10^−6^.

## 3. Results

The main point of this paper is to study spike anticipation as a novel phenomenon that could be taken into account during the study and characterization of information processing in neural networks. In order to support this point we will describe and characterize qualitatively the effects of spike anticipation over main functional aspects of neural networks as their the spiking dynamics and their plasticity.

### 3.1. Spike anticipation in biologically-inspired networks

**Figure 1:**
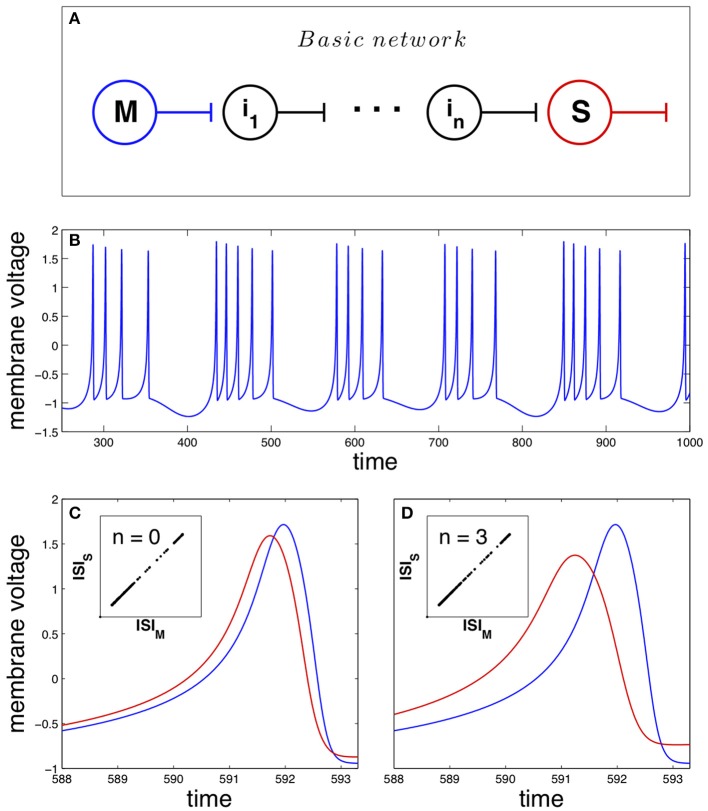
**Anticipating spike synchronization in Hindmarsh–Rose neurons**. **(A)** Basic neural network that will be used in the paper. The master (M) and slave (S) neurons are connected through *n* intermediary neurons (*i*_*j*_ with *j* = 1, …, *n*). All of them are modeled as Hindmarsh–Rose neurons with a specific arrangement of their oscillation frequencies (see Section Materials and Methods for details). **(B)** Chaotic dynamical state (bursting) of the neurons (blue for the master neuron activity). **(C)** With no intermediaries, i.e., direct master-slave coupling, spike anticipation appears (blue line for master and red line for slave), keeping the phase synchronization (inset). **(D)** When three intermediary neurons exist with a proper distribution of their firing frequencies (see text) spike anticipation is up to fourfold enhanced (measured as the difference between the maxima of the closest master and slave action potentials).

As pointed previously, the basic network studied in this work is mathematically described by Equation 1 and depicted in Figure 1A. The master neuron displays a chaotic dynamics structured in bursts (sequences of spikes separated by silent intervals), shown in Figure 1B. The unidirectional coupling of the neurons in the network forces the synchronization of their dynamics, where spike anticipation appears according to the number of intermediary neurons. Figure 1C illustrates this anticipation by showing superimposed two synchronized spikes of the master (blue curve) and the slave (red curve) neurons when no intermediaries are present (*n* = 0); the inset shows the synchronized state by correlating the master and slave interspike intervals or ISI. On the other hand, the introduction of three intermediary neurons (*n* = 3) enhances four-fold the spike anticipation, as shown in Figure 1D, keeping the synchronization of the system (inset).

**Figure 2:**
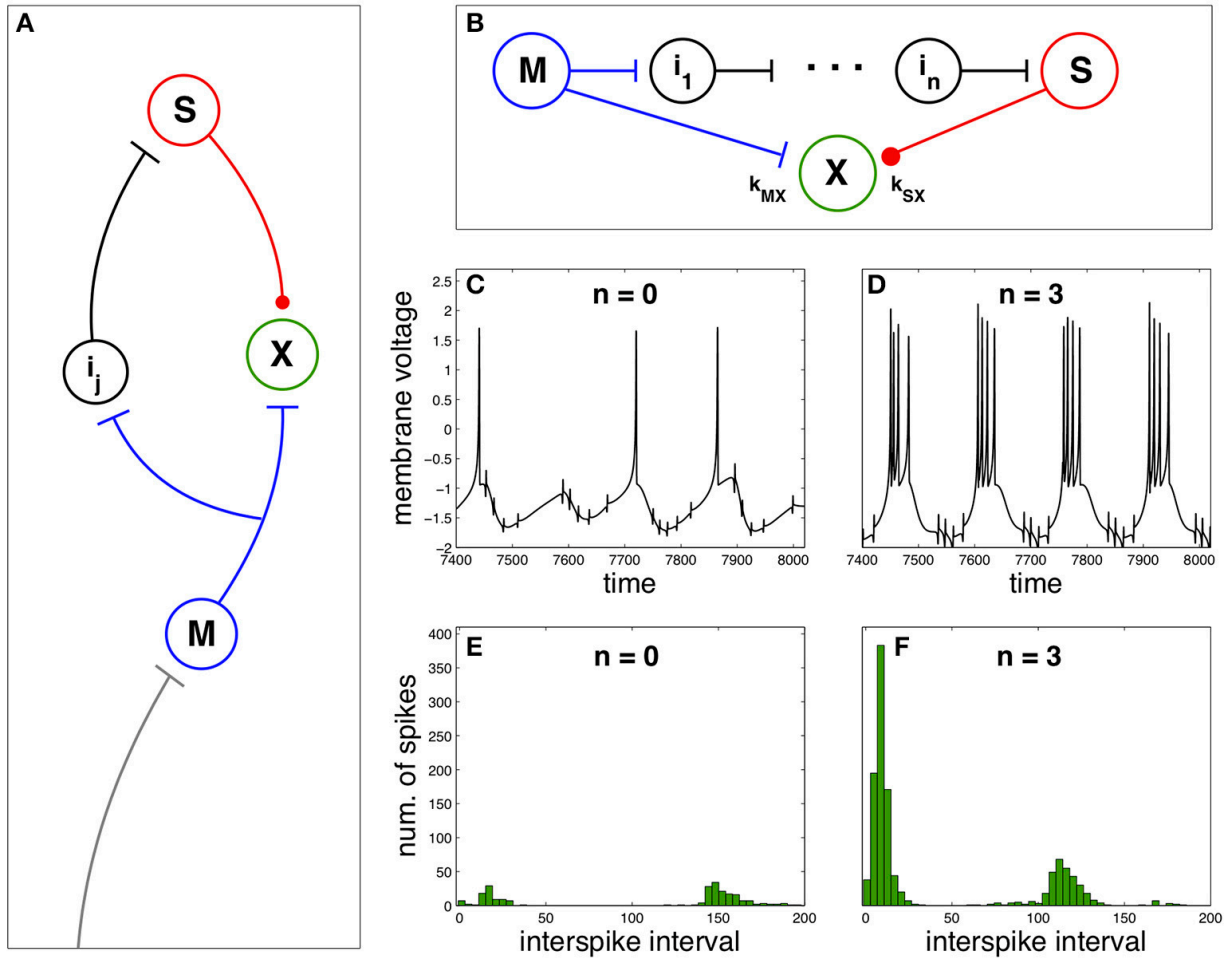
**Firing regimes of a biologically-inspired neural network under spike anticipation. (A)** Neural network where the afferent information is introduced in the master neuron and conveyed to the closed loop where the neuron X is driven by the excitatory (blue flat arrow end) and inhibitory (red dot arrow end) inputs from master and slave neurons. **(B)** Detail of the closed loop, composed of *n* intermediary neurons. We denote the coupling between neuron X and master and slave by *k*_*MX*_ and *k*_*SX*_, respectively. **(C,D)** Chaotic dynamics of the neuron X under different spike anticipation, induced by introducing different intermediaries (*n* = 0 and *n* = 3, respectively). **(E,F)** Statistical analysis of the neuron X activity by means of ISI histograms. Neuron X also follows the Hindmarsh–Rose model described in the Section Materials and Methods adopting the same values for the majority of the common parameters, and with *J*_0*X*_ = 1.3, *C*_*X*_ = 1, *k*_*MX*_ = 3 and *k*_*SX*_ = −3. *T* = 5 × 10^4^.

Now we explore the effect of spike anticipation in a neural network exhibiting diverse characteristics typical of both biological and artificial neural networks, as the presence of closed loops, excitatory and inhibitory synapses (responsible for generating and modulating the network dynamics), relay or intermediate neurons, convergent and divergent flux of information, etc. This network is depicted in Figure 2A, where master (blue) and slave (red) neurons are coupled to a third neuron X (green), whose dynamics will depend on spike anticipation through the master-slave synchronization. The couplings strength of neuron X with master and slave neurons are denoted by *k*_*MX*_ and *k*_*SX*_, respectively, where we will consider positive values for *k*_*MX*_, modeling the excitatory afferent to the neuron X from the master, and negative values for *k*_*SX*_ describing the inhibitory input to X from the slave neuron.

In order to study the influence of spike anticipation over the network dynamics we will pay attention to the behavior of the neuron X. Figures 2C,D show the activity of the neuron X when master and slave neurons are directly coupled (no intermediaries) and when the network contains three intermediary neurons, respectively. The difference in spike anticipation induced by a different number of intermediary neurons (see Figures 1C,D) leads to a significant change in the activity of the neuron X. The quantitative analysis of this activity in both conditions is shown in Figure 2E (*n* = 0) and Figure 2F (*n* = 3), where the histograms of ISI for the neuron X activity (5 × 10^4^ units of time) reveal the appearance of spike bursts (increasing the spiking frequency) and a decrease of the interburst time intervals when spike anticipation increases, being these kinds of spiking activity are main features of neural dynamical behavior observed experimentally in the nervous system. (Steriade et al., 1991).

**Figure 3:**
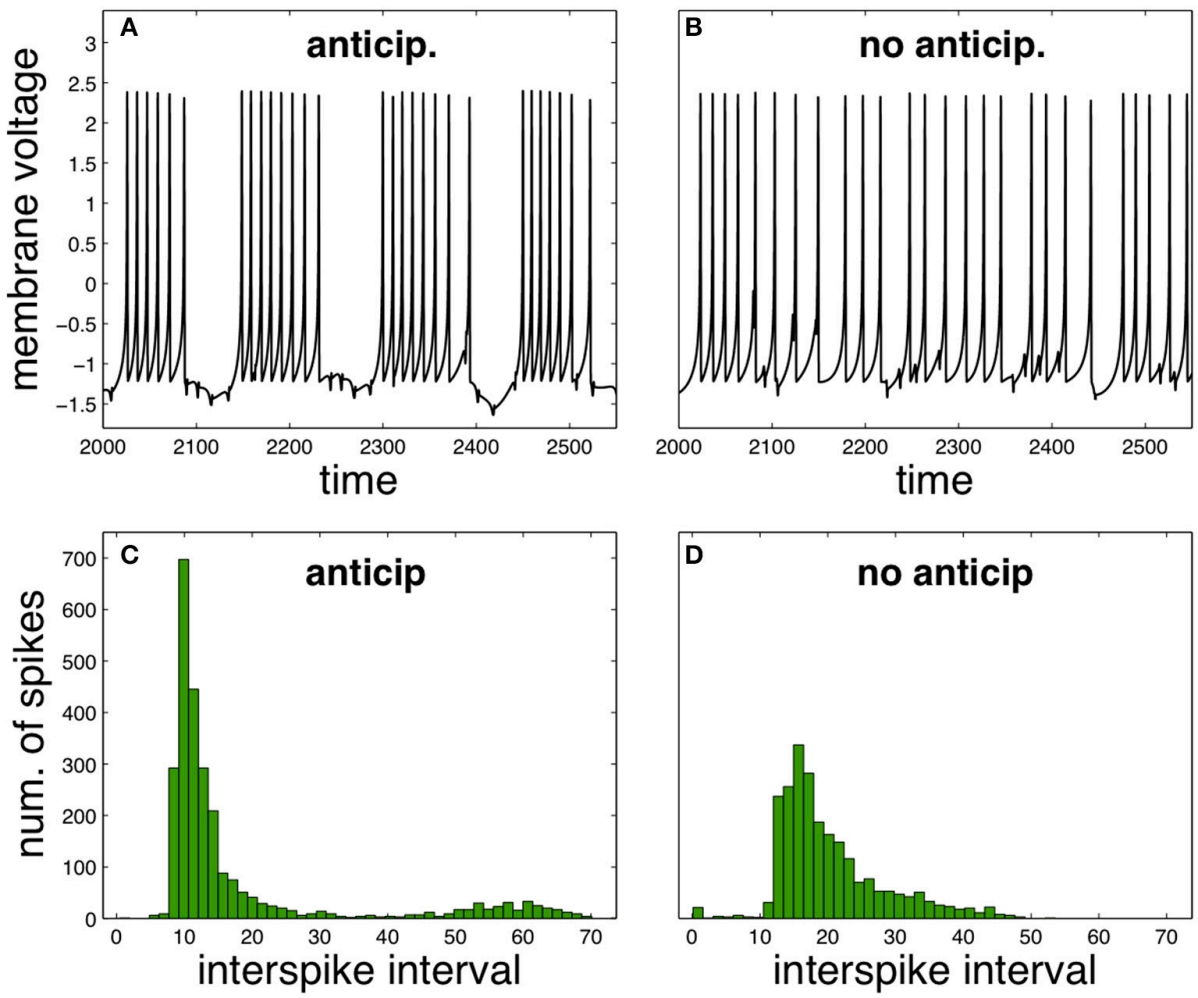
**Comparison of neural activity when anticipation and no anticipation exists**. **(A,B)** Phasic and tonic firing of neuron X under anticipation and no anticipation, respectively. **(C,D)** ISI histograms summarizing these different behaviors. Parameters: *n* = 3, *J*_0*X*_ = 3.25, *C*_*X*_ = 0.7. For the anticipation case *k*_*MX*_ = 0.7 and *k*_*SX*_ = −1 and for the no anticipation case *k*_*MX*_ = −1 and *k*_*SX*_ = 0.7. *T* = 5 × 10^4^.

It is possible to analyse how the functional neural regime can be affected by spike anticipation in a complementary way, by taking advantage from the synchronization between master and slave neurons. Let us consider again the scenario provided by the previous neural network with *n* = 3, analysing the activity of neuron X. In these conditions we consider the state as “anticipation” since synchronization makes the spiking behavior of master and slave neurons equivalent but with the later advanced in time. In consequence we simulate a “no anticipation” state in the same conditions by interchanging the values of the couplings, which will be equivalent to introduce master and slave's signals in the neuron X with no anticipation, i.e., with the slave signal following the evolution of the master one. The results are shown in Figure 3, where Figures 3A,B depict the neuron X activity in anticipation and no anticipation states, respectively, and where Figures 3C,D summarize by ISI histograms. These results reveal how spike anticipation can change dramatically the spiking behavior from tonic spiking to phasic behavior, with the appearance of periodic spike bursts.

**Figure 4:**
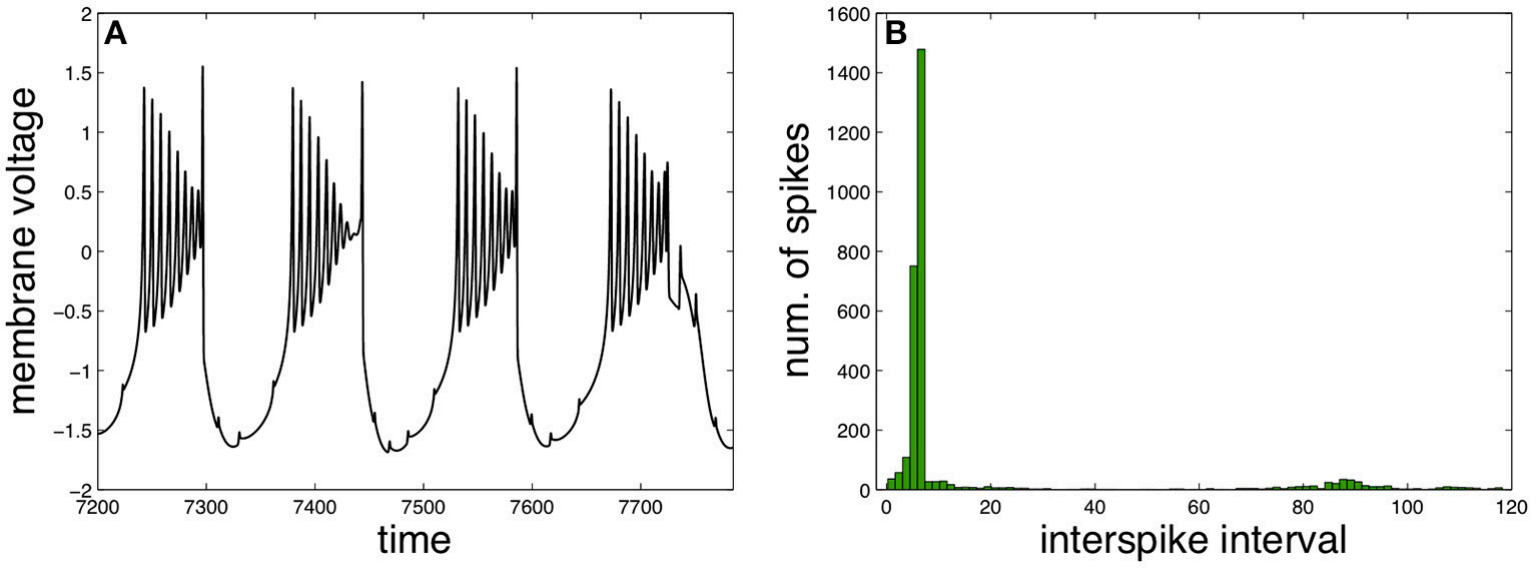
**Fast spiking in slow burst induced by spike anticipation**. **(A)** Neuron X activity. **(B)** Corresponding ISI histogram. Parameters: *n* = 3, *J*_0*X*_ = 3.25, *C*_*X*_ = 1, *k*_*MX*_ = 0.6 and *k*_*SX*_ = −0.3.

A different functional regime for the neuron X appears with three intermediary neurons and by changing the functional parameters properly to elicit a qualitatively different behavior, as illustrated in Figure 4. Such behavior is characterized by slow bursts of fast spikes with decreasing amplitudes, resembling real neural activity observed experimentally in different contexts (Weyand et al., 2001; Viemari et al., 2013).

### 3.2. Discrimination of spikes by anticipation

We have analyzed how the introduction of a different number of intermediary neurons enhances spike anticipation (Figures 1C,D), however this network feature has a deeper impact over the neural information processing since such anticipation will be related to the type of spike that is anticipated. In order to study this point we briefly come back to the initial basic network in Figure 1A, and correlate the ISI of each spike elicited during the activity of the slave neuron with its corresponding spike anticipation.

**Figure 5:**
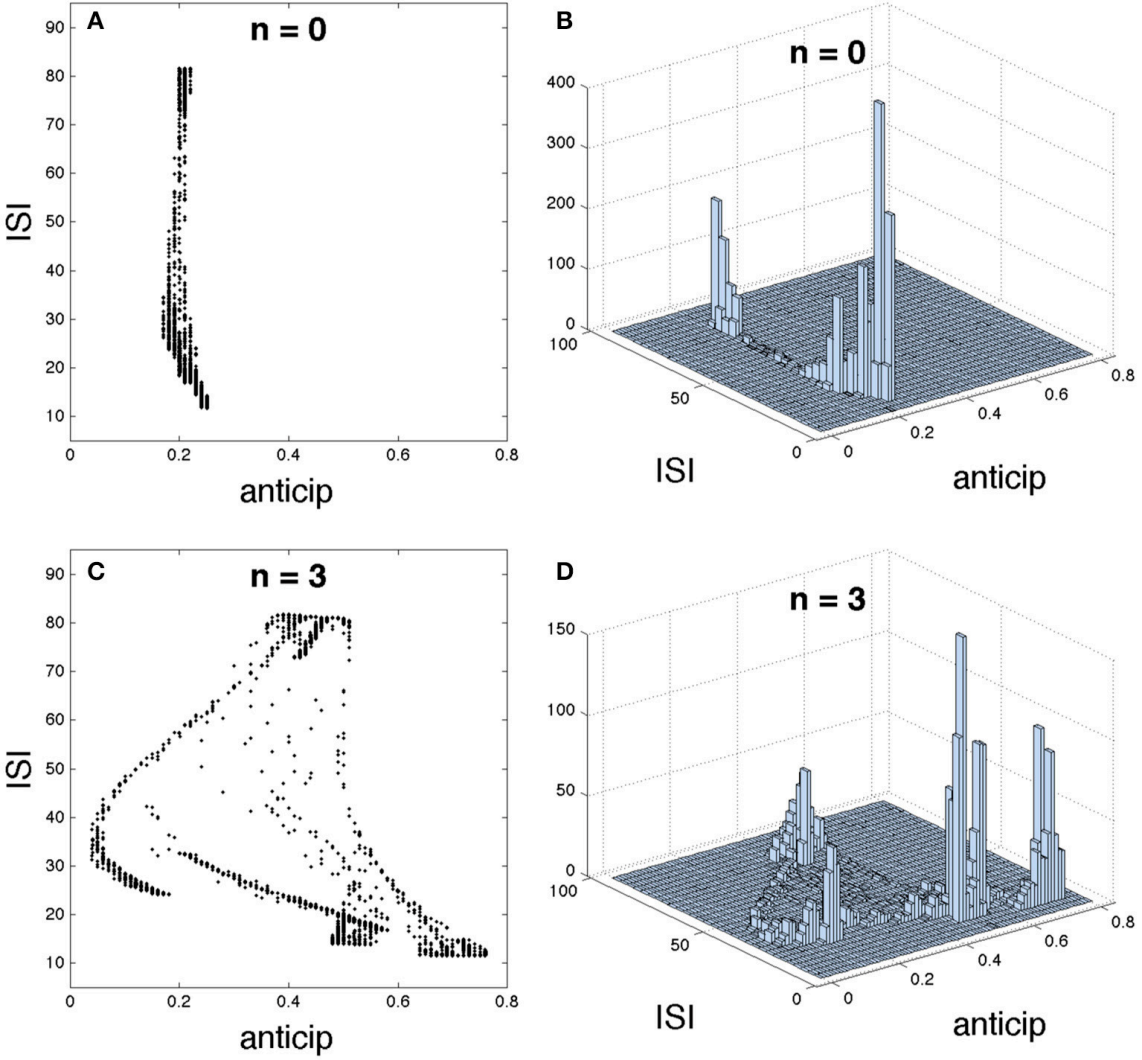
**Detailed structure of the relationship between interspike interval (ISI) and spike anticipation**. The basic neural network in Figure 1A was used as testbed. For *n* = 0 it is shown **(A)** ISI vs. anticipation correlation and **(B)** the 3D histogram, illustrating the spike distribution over the different regions in **(A)**. Panels **(C,D)** show the same respective diagrams for *n* = 3.

The Figure 5 shows such correlation for *n* = 0, i.e., with no intermediary neurons, (upper row) and for *n* = 3 (lower row), by using different graphical representations. The 2D plot in Figure 5A and the 3D histogram in Figure 5B (showing how spikes are accumulated in each region) reveal that a direct coupling between master and slave neurons provides a similar anticipation for every spikes, regardless of their ISIs. However, when master and slave neurons are coupled through three intermediary neurons a complex relationship appears, correlating different spike anticipations with different ISIs as illustrated in Figures 5C,D. In order to interpret this result we must keep in mind the spiking regime exhibited by the slave neuron (Figure 1B; note that this is almost equal to the master behavior since they are synchronized). This neural activity is organized in bursts, containing spikes whose intra-burst ISIs adapt with time, i.e., in the same burst “fast” (low ISI) and “slow” (higher ISI) spikes coexist. When the basic network contains three intermediary neurons it can be seen that: (1) the lowest spike anticipations ([0.05, 0.18]) correspond to the slowest intra-burst spikes, which signalize the end of the burst, (2) significant higher anticipations appear for faster intra-burst spikes, with a clear difference between the fastest spikes (anticipations in [0.67, 0.75]) and the remaining ones (anticipations in [0.49, 0.57]), and (3) medium anticipations ([0.4, 0.48]) correspond to the inter-burst ISIs (highest ISIs), i.e., to those spikes signalizing the beginning of the burst. In consequence these results indicate that specific parts of the neural code conveying different neural information can be discriminated by using their corresponding spike anticipation, suggesting the potential impact of this phenomenon over neural information processing.

### 3.3. Synaptic plasticity under spike anticipation

We illustrate the relation between spike frequency and anticipation by focusing on synaptic plasticity, a critical factor of the network information processing. Let us consider a type of synaptic plasticity based on the reinforcement of the causal relationships between afferent information. This mechanism plasticity tunes the coupling strength between neurons depending on the relative timing or coincidence degree between presynaptic and postsynaptic spikes (Markram et al., 1997, 2012). More in detail, if a presynaptic spike precedes a postsynaptic action potential (elicited by a second input), the corresponding synapse is potentiated, as an enhancement of their possible causal relationship. On the contrary if the presynaptic spike appears after the postsynaptic one, the synapse relating them is depressed, reflecting an unlikely causal relationship among them. Nevertheless, the simplicity of this idea this mechanism is currently considered as one of the main processes in the activity-dependent development of the nervous system.

**Figure 6:**
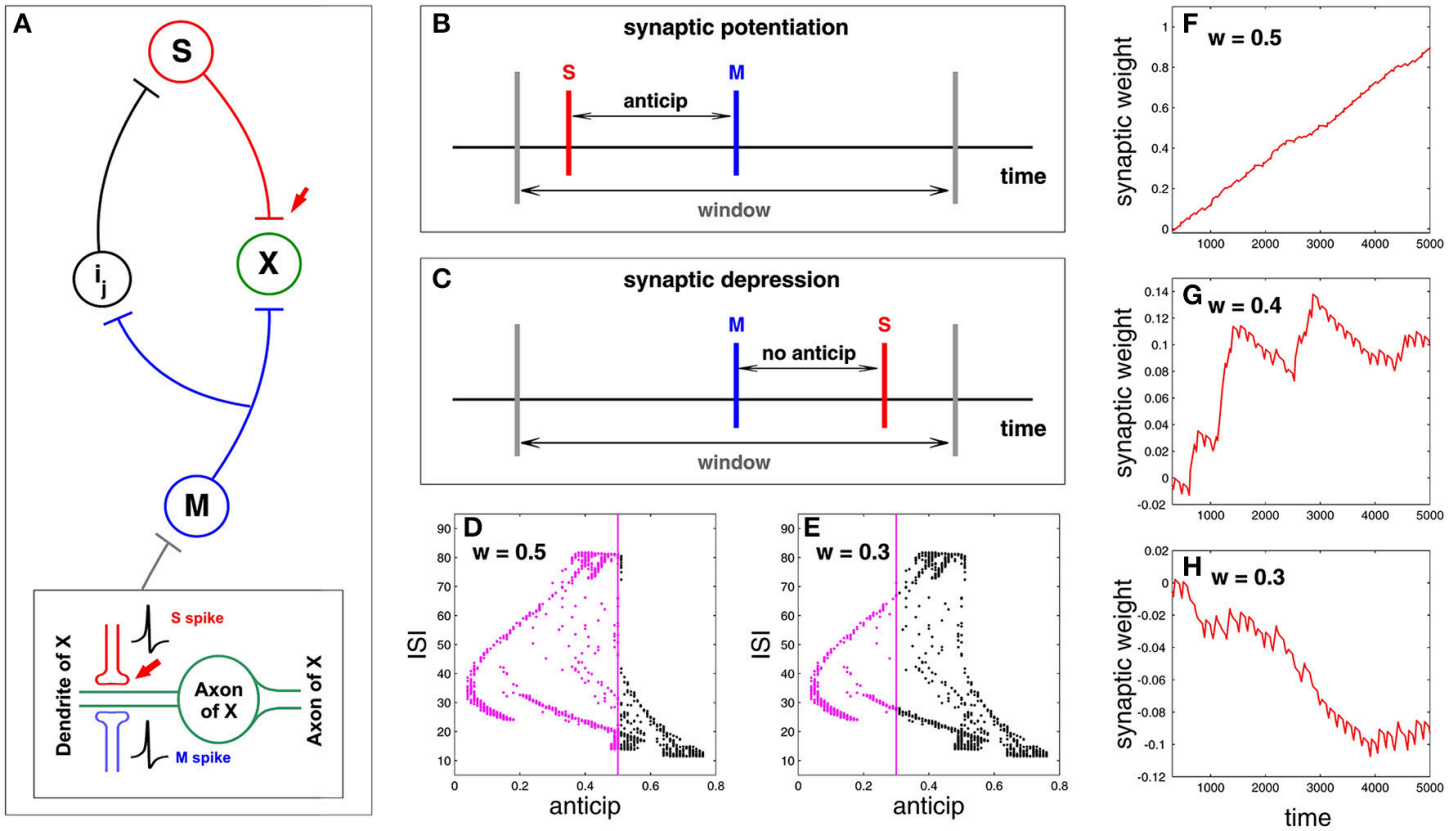
**Synaptic plasticity under spike anticipation**. **(A)** Neural network with a loop composed of three intermediate neurons between master (M) and slave (S), and where information flux converges into the neuron X. Red arrow points the synapse whose plasticity is analyzed by monitoring the evolution of its coupling weight *k*_*SX*_. The bottom inset shows in detail the connection scheme involving the analyzed plasticity. **(B)** Scheme of synaptic plasticity when potentiation appears; the S event (the maximum of the spike received by X neuron from the slave one), denoted by a red vertical line, precedes the M event (blue vertical line) inside the temporal window (gray lines) due to spike anticipation. **(C)** When there is no anticipation the M event precedes the S one and the synapse is depressed. **(D)** The relationship between anticipation and ISI (Figure 5C) reveals that the wider the window, the larger the number of spikes that can coincide inside the window (denoted by magenta points), since the corresponding anticipations lie in the range of the window width. This panel corresponds to *w* = 0.5. **(E)** When *w* = 0.3 the range of anticipations less than or equal to *w* is smaller, so there are fewer spikes that can potentiate the synapse. **(F–H)** Present the time evolution of the synaptic weight *k*_*SX*_ evaluated for three different windows *w* = 0.5, *w* = 0.4, and *w* = 0.3, respectively. The increasing and decreasing rates of the synaptic reinforcement (i.e., the change of the synaptic weight) are *r*_*i*_ = 0.02 and *r*_*d*_ = *r*_*i*_/7, respectively.

Here we study synaptic plasticity under spike anticipation focusing on the previous idea of causal potentiation/depression of neural connectivity. For doing that we study the neural network presented in Figure 2, paying attention to the synapse connecting slave and X neurons (red arrow in Figure 6A) and showing how plasticity can be affected by the complex structure of the relationship between spike anticipation and ISIs (Figure 5). The key factor of the network in Figure 2 is the input provided to X neuron by master (M) and slave (S) neurons since their causal correlation will be the factor for potentiating or depressing the synapse: when the X neuron receives a spike from the S neuron before the spike from the M neuron arrives the S–X synapse is potentiated, being depressed on the contrary. This mechanism is inspired by spike timing dependent plasticity or STDP models (Friedel and van Hemmen, 2008; Butts and Kanold, 2010) with the difference that they focus on the input–output spike correlation and we analyse the causal relationship between spikes of different inputs, assuming that this is the reflection of a causal correlation between input and output. Figure 6B shows a scheme of such a process, where red and blue vertical lines denote the maxima of the S and M spikes, respectively, and gray vertical lines denote the temporal window where both spikes must coincide in the correct order to elicit potentiation. In the studied neural network spike anticipation provides this preceding behavior and so the synaptic potentiation. On the contrary, when no anticipation exists the M spike precedes the S spike and the synapse is depressed (Figure 6C).

The plasticity model is defined by three parameters: the increasing and decreasing rates of the synaptic reinforcement, *r*_*i*_ and *r*_*d*_, respectively [which, for the sake of simplicity and unlike the STDP standard models (Davison and Frégnac, 2006), have been considered non-dependent on the time difference between spikes], and the width *w* of the window where S and M spikes must coincide in the correct order for potentation. The width of the temporal window (characterized by the parameter *w*, where *width* = 2*w*) affects the synaptic potentiation/depression since the wider the window the larger the range of anticipation and the larger the set of spikes reinforcing the synapse. To illustrate this point we represent again the graphic of anticipation vs. ISI (Figure 5C) enlightening in magenta the spikes with an anticipation less than or equal to 0.5 and 0.3, which corresponds to a window *w* = 0.5 and *w* = 0.3 (Figures 6D,E respectively); all these spikes will provide synaptic potentiation, as shown in Figure 6F, whereas a more balanced situation between potentiation and depression takes place for *w* = 0.4 (Figure 6G). In the same way, when *w* = 0.3 there are less spikes whose anticipation lies in the window so the synapse is progressively depressed, as shown in Figure 6H.

## 4. Discussion

### 4.1. Spike anticipation and information processing

As demonstrated by Pyragiene and Pyragas (2013) spike anticipation may appears between two directionally connected neurons under simple conditions. This paper explores how the presence of this spike anticipation in synchronized neural networks can alter their functional regimen, with the subsequent impact over their information processing. In order to analyse this phenomenon we have studied simple neural networks biologically motivated, assuming that information processing in these networks is based on one of the central paradigms in neural coding, which claims that the information is mainly coded in the ISI (Koch, 1999). Therefore, under this relevance of the spike timing, the main objective of this paper is to show that the appearance of spike anticipation could be a novel factor to be taken into account when information processing is studied in both real and artificial neural networks.

We have simulated a basic neural network composed of a variable number of intermediaries connecting the master with the slave neuron, mathematically described by a set of coupled Hindmarsh–Rose neurons. This network has been configured as a minimal model to capture the functional essence of a typical neural network of the mammal nervous system (distant afferents, closed loops, intermediary neurons as relay stations, convergent/divergent information flow, etc.). The numerical simulations performed with this model suggests that the existence of spike anticipation can induced the appearance of a diversity of qualitatively different behaviors: (1) firing of distant single spikes as seen at both thalamic and cortical anticipatory activity during active tactil discrimination (Pais-Vieira et al., 2013), bursting of slow and fast spikes, phasic spiking and tonic firing which resemble state-dependent changes in thalamic firing (i.e., between sleep and wakefulness Tsoukatos et al., 1997; Fanselow et al., 2001; Weyand et al., 2001). Such behaviors have been observed as functional patterns associated to different types of information processing in real neurons.

A more detailed analysis of the relationships between spike anticipation and the structure of the action potential sequences induced by the synchronization of master, intermediary and slave neurons, has been performed. It reveals a deeper correlation between the preceding time of a slave spike with respect to the master activity and the ISI of this spike. Therefore, spike anticipation would allow to discriminate different types of action potentials, conveying different types of information, as those defining the duration of the burst, the time interval between consecutive bursts, the inter-burst information, etc. A real scenario where this discrimination could be exploited is the synaptic plasticity by potentiation-depression of the synapse between the slave neuron which receives directly input from the master. In these conditions the synapse will be potentiated only if a S spike precedes a M spike in a certain time window, depressing it in other case. Our results show that under different windows distinct anticipations appears between S and M spikes, and a variable percentage of them affect to the reinforcement of the synapse, changing the synaptic plasticity in different ways.

This dynamical effect would have a significant impact over the neural network since (1) the potentiation-depression of a synapse implies the causal strengthening/debilitation of the conveyed information through this connection, and (2) the sensitivity of a synapse to transmit information is a key factor of the processing capability of the network (Swadlow et al., 2005; Daw et al., 2007; Khazipov et al., 2013). Living out of the synaptic delay (see next section), the plasticity mechanism here studied is inspired by the Spike-Timing-Dependent Plasticity or STDP observed in many synapses in real neural networks, as those in the thalamocortical loop, where the sensory information can be potentiated/depressed according to the temporal relationship between spikes coming from the cortex (Daw et al., 2007; Grossberg and Versace, 2008; Sanchez-Jimenez et al., 2013). Although, this work studies the impact of STDP-inspired plasticity, obviously real neuronal network plasticity will depend on others well known plasticity mechanisms such as the contribution of individual spikes from complex spike patterns, the presence of presynaptic spike bursts, dendritic location, the existence of other inputs, excitability changes, synaptic competence and so on (Artola and Singer, 1993; Sjostrom et al., 2001; Froemke and Dan, 2002; Hausser and Mel, 2003; Lin et al., 2003; Caporale and Dan, 2008; Fiete et al., 2010).

### 4.2. Model limitations and future work

Neural models require a simplified description due to the functional and structural complexity of real neurons, which makes unattainable an exact and rigorous analysis. Such simplifications demand a subtle balance between accuracy and accessibility to capture their functional essence but ensuring an affordable capability of description.

The present study, focused on spike anticipation on the context of neural information processing, is based on a mathematical model that does not consider a variety of aspects of real neural networks. On the one hand the first question is how the particular model used to describe the neurons could affect the obtained conclusions. However, it is shown in Pyragiene and Pyragas (2013) that this anticipation in synchronized systems appears in a variety of different oscillatory systems, including distinct neural models. Therefore, this kind of anticipation seems to be a property of the dynamics of the system, with no strong dependency on its particular description.

On the other hand there exist a critical aspect in real neurons concerning the intrinsic delay in the generation and transmission of information in real neural networks. The two main factors that introduce a significant delay during the operation of real neural networks are the finite velocity of action potential propagation and especially the information transmission through chemical synapses (Koch, 1999; Kandel et al., 2000). Actually this delay, together with feedback connections, has been proposed as one mechanism to induce anticipating synchronization. However, the anticipation mechanism studied here does require less restrictive conditions and the impact of the delay associated to the chemical network couplings over spike anticipation will be tackled in a future work. Nonetheless, even in the case when no pure master-slave anticipation would occur, such anticipation could appear in a *reduction* of the effective delay in the network, which is a critical parameter associated to information processing of the network (Sanchez-Jimenez et al., 2009).

Another relevant issue concerns the plausibility of the condition over the spiking frequencies of the neurons. When just two neurons are coupled it is obviously likely that the driven neuron could be faster than the driver one. When the network is compound of five neurons (three intermediaries) the same relation is required for each pair of coupled neurons, but the qualitative spike anticipation (and its enhancement) is robust with the distribution of spiking frequencies, beyond the equally-distributed frequencies here used by simplicity (data not shown).

In summary in this paper, through simple but biologically-motivated scenarios, we have shown how spike anticipation could have a significant influence over neural networks both at functional level, considering the neural firing regimes and the different information coding that they imply, and at structural level, redefining key activity-dependent features of the network. This results suggest that spike anticipation in neural networks exhibiting non-trivial structures (distant afferents, closed loops, intermediary neurons, etc.) could be a novel factor to be considered together with other well-known processes, input excitatory/inhibitory balance, time-delay, spiking regime of individual neurons, network topology, etc.

## Author contributions

DS: simulations and data analysis; AS: design of the work and text writing; MG: simulations and data analysis; AN: simulations and data analysis; JV: design of the work, simulations, data analysis, and text writing.

### Conflict of interest statement

The authors declare that the research was conducted in the absence of any commercial or financial relationships that could be construed as a potential conflict of interest.
